# Early prediction of pathological response to neoadjuvant chemotherapy of breast tumors: a comparative study using amide proton transfer-weighted, diffusion weighted and dynamic contrast enhanced MRI

**DOI:** 10.3389/fmed.2024.1295478

**Published:** 2024-01-17

**Authors:** Nan Zhang, Qingwei Song, Hongbing Liang, Zhuo Wang, Qi Wu, Haonan Zhang, Lina Zhang, Ailian Liu, Huali Wang, Jiazheng Wang, Liangjie Lin

**Affiliations:** ^1^Department of Radiology, First Affiliated Hospital, Dalian Medical University, Dalian, China; ^2^Department of Radiology, Zhongshan Hospital, Fudan University, Shanghai, China; ^3^Department of Pathology, First Affiliated Hospital, Dalian Medical University, Dalian, China; ^4^MSC Clinical and Technical Solutions, Philips Healthcare, Beijing, China

**Keywords:** amide proton transfer weighted, neoadjuvant chemotherapy, major histologic responder, breast cancer, protein

## Abstract

**Objective:**

To examine amide proton transfer-weighted (APTw) combined with diffusion weighed (DWI) and dynamic contrast enhanced (DCE) MRI for early prediction of pathological response to neoadjuvant chemotherapy in invasive breast cancer.

**Materials:**

In this prospective study, 50 female breast cancer patients (49.58 ± 10.62 years old) administered neoadjuvant chemotherapy (NAC) were enrolled with MRI carried out both before NAC (T0) and at the end of the second cycle of NAC (T1). The patients were divided into 2 groups based on tumor response according to the Miller-Payne Grading (MPG) system. Group 1 included patients with a greater degree of decrease in major histologic responder (MHR, Miller-Payne G4-5), while group 2 included non-MHR cases (Miller-Payne G1-3). Traditional imaging protocols (T1 weighted, T2 weighted, diffusion weighted, and DCE-MRI) and APTw imaging were scanned for each subject before and after treatment. APTw value (APTw0 and APTw1), Dmax (maximum diameter, Dmax0 and Dmax1), V (3D tumor volume, V0 and V1), and ADC (apparent diffusion coefficient, ADC0 and ADC1) before and after treatment, as well as changes between the two times points (ΔAPT, ΔDmax, ΔV, ΔADC) for breast tumors were compared between the two groups.

**Results:**

APT0 and APT1 values significantly differed between the two groups (*p* = 0.034 and 0.01). ΔAPTw values were significantly lower in non-MHR tumors compared with MHR tumors (*p* = 0.015). ΔDmax values were significantly higher in MHR tumors compared with non-MHR tumors (*p* = 0.005). ADC0 and ADC1 values were significantly higher in MHR tumors than in non-MHR tumors (*p* = 0.038 and 0.035). AUC (Dmax+DWI + APTw) = AUC (Dmax+APTw) > AUC (APTw) > AUC (Dmax+DWI) > AUC (Dmax).

**Conclusion:**

APTw imaging along with change of tumor size showed a significant potential in early prediction of MHR for NAC treatment in breast cancer, which might allow timely regimen refinement before definitive surgical treatment.

## Introduction

Neoadjuvant chemotherapy (NAC) has become the standard treatment option for locally advanced breast cancer. Early and accurate prediction of tumor response to NAC is critical for treatment management (1, 2). However, breast cancer’s response to NAC varies widely among different patients, it is estimated that 19–30% of patients experience major histologic responders (MHRs) and 5–20% exhibit non-major histologic responders (non-MHRs) (3, 4). The 2019 National Comprehensive Cancer Network (NCCN) guidelines for breast cancer suggest magnetic resonance imaging (MRI) may help assess tumor range, remission status after treatment and feasibility of breast-conserving surgery before and after NAC (5). Functional and molecular imaging MRI methods, including dynamic contrast enhanced MRI (DCE-MRI) (6), intravoxel incoherent motion (IVIM) (7), diffusion kurtosis imaging (DKI) (8), and magnetic resonance spectroscopy (MRS) (9), provide insights into the underlying pathophysiology of tissues from morphology to cellular metabolism (10–12). But, currently, there is still no standard method or imaging biomarker in clinical practice to accurately predict predicting pCR to NAC in patients with breast cancer.

Chemical exchange saturation transfer (CEST) imaging is a novel magnetic resonance molecular imaging method derived from the magnetization transfer (MT) technology (13). It provides molecular level data instead of microstructural information compared to DCE- and DWI-based MR techniques, with orders of magnitude higher detection sensitivity compared to MRS. Amide proton transfer weighted (APTw) imaging, as a kind of CEST method, can reflect the concentration of exchangeable amide protons in endogenous mobile proteins or polypeptides in the cytoplasm, and hence indirectly detects *in vivo* changes in protein expression rate and related pathophysiological features in living cells (14, 15) Dula et al. (16) firstly established breast APTw imaging with good stability and repeatability. In addition, the effect of the menstrual cycle on APTw imaging in human breast was also assessed (17). Our previous study (18) has shown the potential of breast 3D APTw imaging in differetiation between benign and malignant tumors. APTw has also been used to evaluate lymphatic damage and interstitial protein accumulation in patients with breast cancer treatment-related lymphedema (19).

Therefore, we hypothesis that APTw imaging may be a potential tool for assessing the response of breast cancer to chemotherapy, expecially, the early response to NAC (16). Krikken et al. (20) assessed noninvasive early detection of treatment response in 9 patients with breast cancer to NAC using APTw at 7 T. While higher-field strength could improve APTw sensitivity, the associated safety issues cannot be overlooked (21). This study aims to explore whether APTw imaging at 3 T could be used for early evaluation of the efficacy of NAC in breast cancer.

## Materials and methods

### Patients

**Figure 1:**
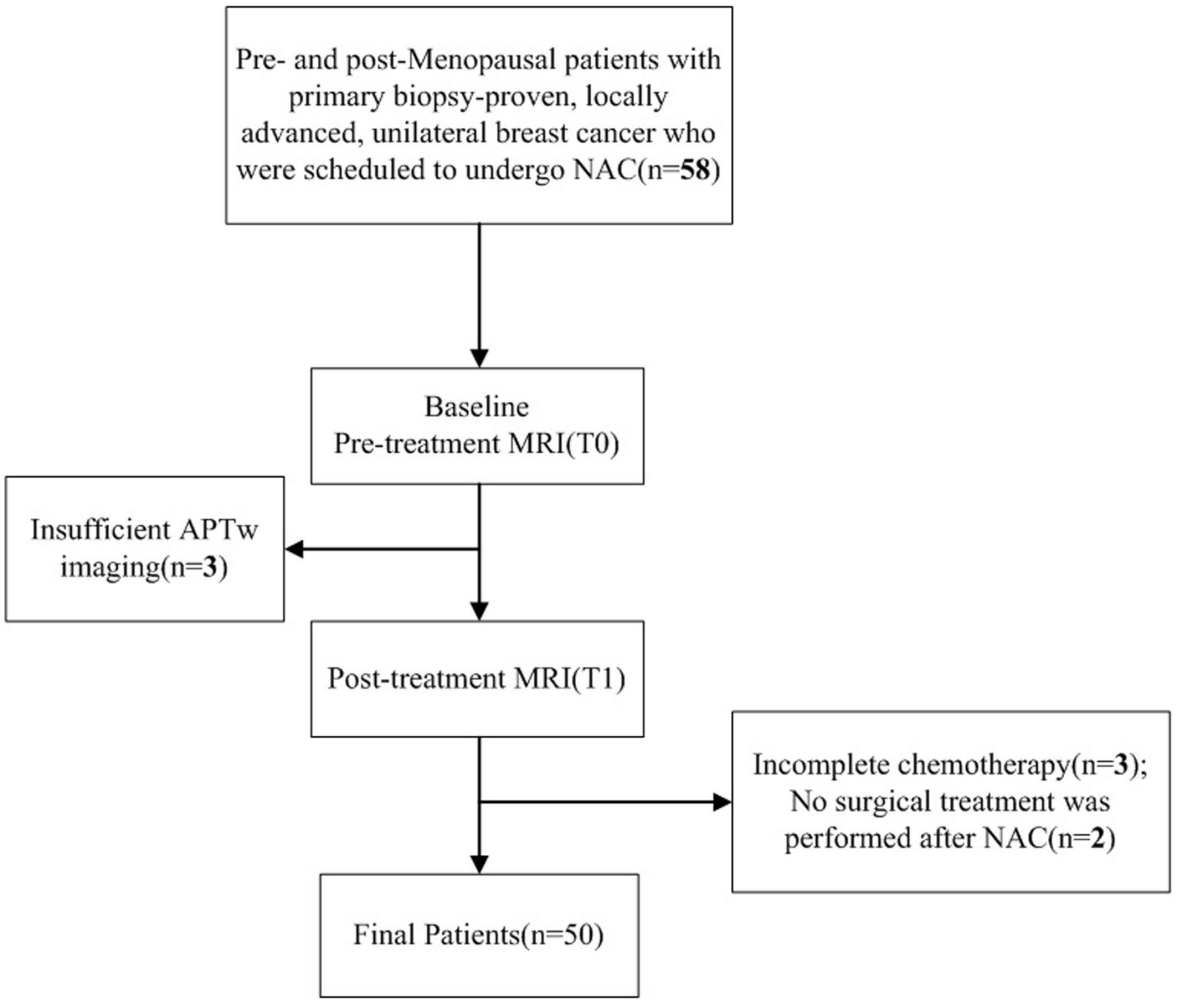
Study flowchart.

This prospective study was approved by the Ethics Committee of First Affiliated Hospital of Dalian Medical University (PJ-KS-XJS-2020-19). Informed consent was obtained from each patient. Patients with primary biopsy-proven, locally advanced, unilateral breast cancer scheduled for NAC between 2020 and 2022 were included. All eligible patients underwent two MR scans, 3–7 days before NAC (T0) and at the end of the second NAC cycle (T1). Totally 58 female patients were initially scheduled for MRI, among whom 50 (mean age, 49.58 ± 10.62 years; age range, 31–68 years) were finally enrolled with the following exclusion criteria: (1) incomplete chemotherapy (*n* = 3); (2) no surgical treatment after NAC (*n* = 2); (3) sub-quality APTw imaging (*n* = 3) (Figure 1).

### MRI protocol

MRI was performed on a 3.0 T scanner (Philips Ingenia CX, Philips Healthcare, the Netherlands) using a seven-channel bilateral phase-arrayed breast coil. Each patient was placed in the prone position without compression of breasts. Each patient was placed in the prone position without compression of breasts, and a sandbag was placed on the patient’s back during the scan to minimized the respiratory movement distortion.APTw images were acquired with a three-dimensional (3D) turbo-spin-echo (TSE) sequence besides traditional imaging protocols (T1WI, T2WI, DWI, and DCE imaging). The applied imaging parameters are detailed in Table 1. The total scanning time is 22 min: 44 s. For APTw imaging (18), the saturation pulse train with a duration of 2 s was applied with 2-μT B1 amplitude at each of the following 6 frequencies for the reconstruction of the Z spectrum in each image voxel: ±2.7 ppm, ±3.5 ppm, and ± 4.3 ppm, where 0 ppm was water proton resonance. A reference acquisition was carried out with a radio frequency (RF) of −1,560 ppm. Three acquisitions were performed at a saturation frequency of +3.5 ppm with shifted echo times to build a B0 map for voxel-wise frequency correction to the Z spectrum. To reduce the influence of B1 field inhomogeneity, unilateral APTw imaging of only one breast instead of two breasts was implemented; to reduce image artifacts induced by chest motion, the prone position was adopted (only data from 2 patients were excluded due to unacceptable motion artifacts). APTw and DWI sequences were conducted before injection of the gadolinium contrast agent (Gadodiamide, Bayer AG). Traditional imaging protocols were prescribed to cover the entire bilateral breast tissue, and the APTw imaging protocol was prescribed unilaterally to cover the entire tumor based on T2WI scans.

**Table 1:** Acquisition parameters of scan sequences.

	T1WI	T2WI	T2WI	DWI	APTw	DCE
Orientation	Tra	Tra	Sag	Tra	Tra	Tra
TR [ms]	653	3,840	3,840	6,500	6,445	4
TE [ms]	8.4	100	100	79	7.8	2
FOV [mm^2^/mm^3^]	281 × 340	281 × 340	240 × 200	300 × 369	130 × 130 × 49	240 × 299 × 160
Voxel size	0.80 × 0.79	0.80 × 0.90	0.80 × 0.90	2.40 × 2.40	2.03 × 2.00 × 7.00	1.00 × 1.20 × 4.00
Slice thickness [mm]	4	4	4	4	7	4
Flip angle [°]	90	90	90	90	90	15
TSE factor	14	15	15	--	100	--
Acceleration factor	SENSE 4	SENSE 4	SENSE 4	SENSE 4	SENSE 1.6	CS 4
*b*-value [s/mm^2^]	--	--	--	01000	--	--
Fat suppression	--	SPAIR	SPAIR	SPAIR	SPIR	SPAIR
Bandwidth (Hz/pixel)	221.9	218.2	218.0	31.8	702.5	826.7
Saturation pulse/duration	--	--	--	--	2.0 μT, 200 ms, 4	--
Scan time (min: sec)	1:05	3:36	2:21	2:53	4:58	7:51

DCE, dynamic contrast enhanced; SENSE, sensitivity encoding; CS, compressed sensing; SPAIR, spectral attenuated inversion recovery; SPIR, spectral pre-saturation with inversion recovery.

### Image analysis

**Figure 2:**
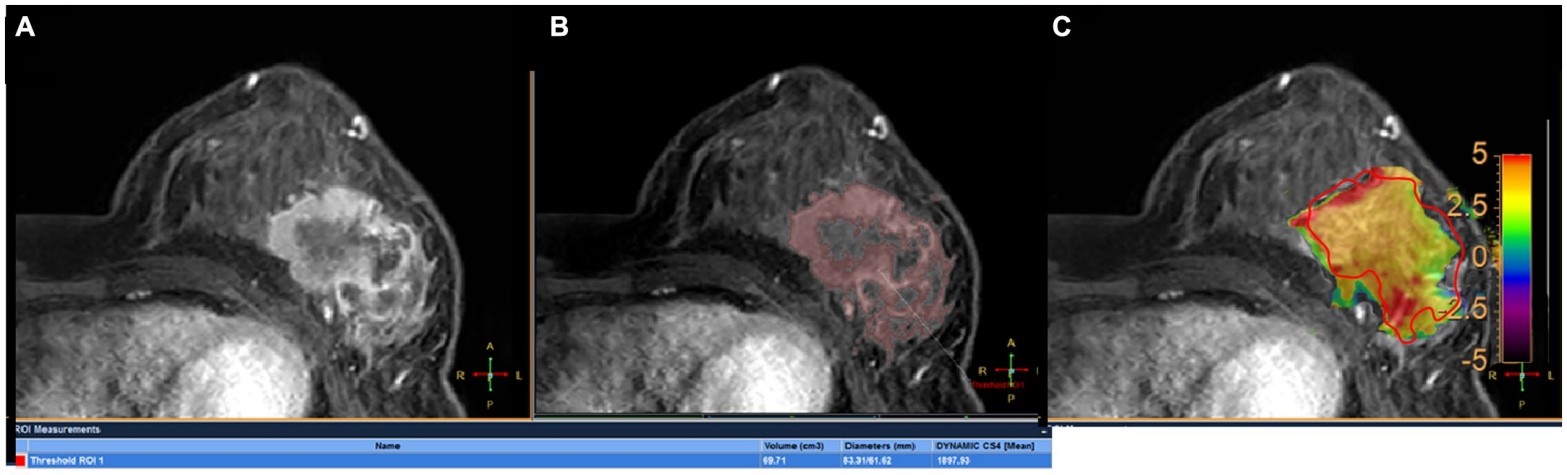
A 45-year-old woman with grade III invasive ductal breast cancer: a 43-year-old woman with grade III invasive breast cancer: **(A)** image of DCE, **(B)** the volume of interest was also determined on the contrast enhanced image using a threshold method, and the Dmax and V measured for tumor were 83.31 mm and 69.71 mm^3^, respectively. **(C)** the tumor region of interest was determined on the contrast enhanced image and copied to the APTw image for APTw value measurement (APTw = 3.51 ± 1.23).

All images were analyzed with the IntelliSpace Portal (ISP, Philips Healthcare, Cleveland, OH, United States) workstation. The magnetization transfer ratio with asymmetric analysis at +3.5 ppm (MTRasym [+3.5 ppm]) for each image voxel was carried out to generate APTw maps from raw images in real time on the console with *Z*-spectrum fitting and B0 correction (14), where values in the maps refer to differences between the signal intensities at ±3.5 ppm of water proton resonance, as percentages of the signal intensities when the saturation pulse applied is far off resonance. Image analysis was carried out by two breast radiologists (readers 1 and 2 with 13 and 6 years of experience in imaging diagnosis, respectively, blinded to final pathological results and other clinical data). In each patient, APTw maps were firstly fused onto DCE images at approximately the same slice position (22), and a 2D region of interest (ROI) was delineated on the slice transecting the largest area of the lesion. The ROIs included the most enhanced lesion regions on DCE images while avoiding cystic or necrotic lesions (Figure 2A). The maximum diameter (Dmax) and 3D volume (V) of each tumor were measured on DCE images. The threshold extraction method of the MR Segmentation software on the workstation (Intellispace Portal v7.0, Philips Healthcare) was used to extract the tumor as a whole, and the software automatically yielded Dmax and V (Figures 2B,C). Apparent diffusion coefficient (ADC) values were measured on DWI images. APTw, V, Dmax and ADC values at the time points T0 and T1 were annotated as APTw0, APTw1, V0, V1, Dmax0, Dmax1, ADC0 and ADC1, and changes in APTw, V, Dmax and ADC values at T1 relative to T0 were termed ΔAPTw, ΔV, ΔDmax and ΔADC, respectively.

Time–intensity curves (TICs) were obtained from DCE images, which were divided into three categories (23): I-type: slow or medium wash-in (0 < SI < 100% increase) in the initial phase and plateau (SI ± 10% change) or persistent (SI < 10% increase) in the delayed phase; II-type: rrapid wash-in in the initial enhancement phase and plateau in the delayed enhancement phase; and III-type: rapid wash-in (SI > 100% increase) in the initial phase and rapid wash-out (SI > 10% decrease) in the delayed phase. Based on morphological features in DCE images, the tumors were classified based on the following criteria (24): mass shape (oval, round and irregular); mass margin (circumscribed and not circumscribed); and internal enhancement (homogeneous, heterogeneous and rim enhancement).

### Pathological analysis

All diagnoses were confirmed by surgical histopathology after MRI. Cancer grades were evaluated based on pathological criteria: grade I, well-differentiated tumor; grade II, moderately differentiated tumor; and grade III, poorly differentiated tumor. The Miller-Payne Grading (MPG) system (Supplementary Table S1) was utilized to assess tumor response (25). MPG 4–5 case were classified as major histologic responders (MHRs), and MPG 1–3 cases were classified as non-major histologic responders (non-MHRs) (26). The final surgical specimen (lumpectomy versus mastectomy) was employed to examine MHRs (Group 1) or non-MHRs (Group 2). Estrogen receptor (ER) and progesterone receptor (PR) positivity was reflected by expression of the given receptor in 10% or more of tumor cells. The expression criteria for human epidermal growth factor receptor-2 (HER2) were negative (+ and – signals) or positive (+++ signals). Samples with ++ signals were further submitted to *in situ* hybridization. Cases with amplified genes were considered positive, and those without amplified genes were considered negative. The cut-point between ‘high’ and ‘low’ values for Ki-67 was 20%.

### Statistical analysi*s*

SPSS (version 21, SPSS Inc., Chicago, IL, United States) was used for data analysis. Intraclass correlation coefficients (ICCs) and the Bland–Altman analysis were utilized to evaluate measurement consistency between the two readers and assess the 95% limit of agreement (95% LoA). An ICC above 0.75 indicated good agreement. The Kolmogorov–Smirnov test was caried out to assess differences in Dmax, V, APTw and ADC values between the two groups. Data are mea*n* ± standard deviation. The Kappa test was carried out to assess differences in TIC type between the two groups. Finally, multivariate logistic regression analysis was conducted for independent variables based on indicators with statistical differences between the two groups. Receiver operating characteristic (ROC) curves were employed to assess the diagnostic performances of various parameters in breast cancer. The DeLong test was performed to analyze differences in areas under the curves (AUCs) for various parameters.

## Results

### Clinicopathological data

Fifty patients (mean age 49.58 ± 10.62 years, ranging from 31 to 68 years) were finally included, whose clinical characteristics are summarized in Table 2. All patients received NAC with types including AC-TH [Adriamycin (A) or epirubicin (E) + cyclophosphamide (C) 4 cycles followed by docetaxel or paclitaxel + herceptin (H) 4 cycles], TCbHP [Taxoid drugs (T), carboplatin double chemotherapy (Cb) + trastuzumab (H), pertuzumab (P) 6 cycles], TEC [Taxoid drugs (T) + epirubicin (E) + cyclophosphamide (C) 6 cycles] shown in Table 2.

**Table 2:** Patient baseline characteristics in the MHR and non-MHR groups.

	All (*n* = 50)	MHR (*n* = 14)	n-MHR (*n* = 36)	*P*
**Mean age (range)**	50 (31–68)	52 (34–68)	49 (31–68)	0.854
**Tumor histologic type**				0.941
Invasive ductal	49	14	35	
Invasive lobular	1	0	1	
**Tumor size pre-NAC**				0.546
T1	3	0	3	
T2	29	8	21	
T3/4	18	6	12	
**N stage pre-NAC**				0.654
N0	16	5	11	
N1	34	9	25	
**Grade**				
I	1	0	1	0.423
II	33	7	26	
III	16	7	9	
**Receptor status**				0.564
ER positive/PR positive	38	10	28	
ER negative/PR negative	12	4	8	
HER2 negative	28	1	27	
HER2 positive	22	13	9	
Triple negative	5	1	4	
**Types of NAC**				0.856
AC-TH	5	2	3	
TCbHP	17	7	10	
TEC	28	5	23	

### Comparison of morphological changes under different NAC responses

In consistency analysis between the two readers, ICC values for data measurements showed high reliability, shown in Table 3. (All ICC > 0.9). Bland–Altman plots also showed excellent consistency between the two groups. Measurements by both readers are listed in Supplementary material, and the data obtained by the senior observer (reader 1) were selected for follow-up analysis.

**Table 3:** Consistency analysis between the two readers for data measurements.

Variable	Reader 1	Reader 2	ICC
APT0 (%)	3.66 ± 0.50	3.78 ± 0.45	0.940
APT1 (%)	2.23 ± 0.86	2.56 ± 0.77	0.921
Dmax0 (mm)	43.02 ± 20.61	43.66 ± 20.99	0.997
Dmax1 (mm)	30.41 ± 16.91	30.56 ± 16.45	0.940
V0 (mm^3^)	28.26 ± 30.90	28.89 ± 30.59	0.992
V1 (mm^3^)	5.44 ± 3.17	5.45 ± 3.78	0.996
ADC0	1.12 ± 0.17	1.55 ± 0.17	0.940
ADC1	1.05 ± 0.11	1.05 ± 0.77	0.921

The two groups did not significantly differ in mass enhancement characteristics (lesion shape, *p* = 0.872; internal enhancement in the initial phase, *p* = 0.544; internal enhancement in the delayed phase, *p* = 0.329; TIC type, *p* = 0.836; edge, 0.971). Likewise, axillary lymph node metastasis (with or without) was not significantly different between the two groups (*p* = 0.728) (Table 4).

**Table 4:** conventional MRI data for patients.

Variable	Total (*n* = 50)	n-MHR (*n* = 36)	MHR (*n* = 14)	*P*
**Lesion shape**				0.872
Oval	1	1	0	
Round	3	2	1	
Irregular	46	33	13	
**Internal enhancement in initial phases**				0.544
Homogeneous	3	3	0	
Heterogeneous	37	26	11	
Rim enhancement	10	7	3	
**Internal enhancement in delayed phases**				0.329
Homogeneous	6	6	0	
Heterogeneous	33	23	10	
Rim enhancement	11	7	4	
**Axillary lymph node metastasis**				0.728
Yes	34	25	9	
No	16	11	5	
**Type of TIC**				0.836
I	33	24	9	
II	11	8	3	
III	6	4	2	
**Margin**				0.971
Circumscribed	7	5	2	
Not circumscribed	43	31	12	

### Comparison of APTw, Dmax, V, and ADC changes under different NAC responses

**Figure 3:**
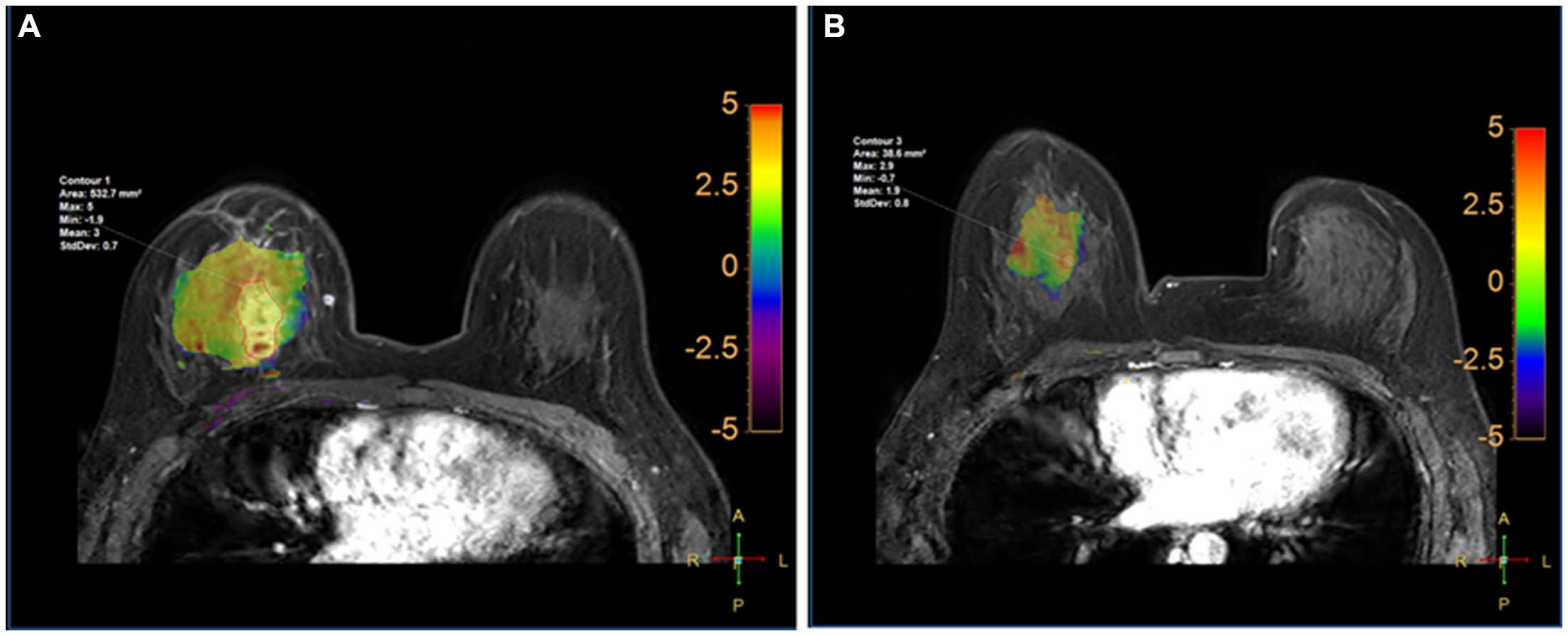
Fusion of APTw and DCE-MRI of the breast of a 45-year-old perimenopausal woman with right Her-2+ invasive breast cancer at T0 **(A)** and T1 **(B)**. The measured APTw0 and APT1 values were 3 and 1.8%, respectively. The case was confirmed by pathological analysis of the surgical specimen collected after the first 2 cycles of NAC as having non-pCR.

**Figure 4:**
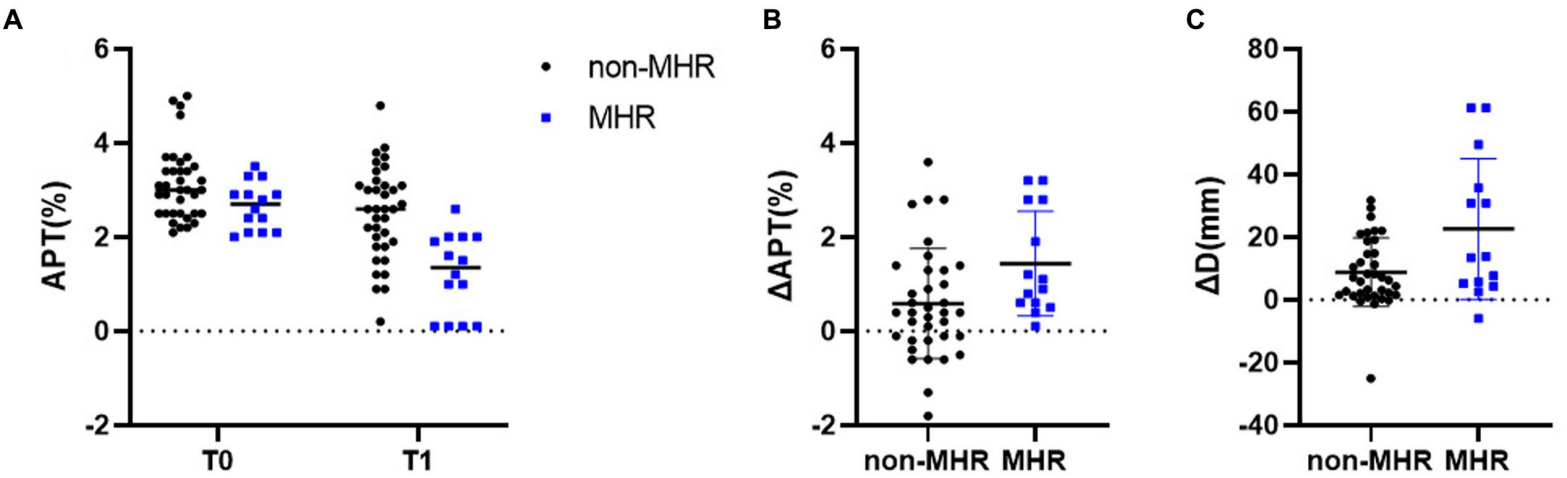
**(A)** APTw values for pathologic non-MHR and MHR groups at baseline (T0), and after two cycles (T1) in the 50 examined participants. Differences in ΔAPT and ΔDmax for the pCR and non-pCR groups are shown in **(B,C)**, respectively. **p < 0.01.

Comparison of APTw between the two groups is shown in Table 5. There were significant differences in APT0 and APTw1 between the two groups (*p* = 0.034, and *p* < 0.001). APTw values of breast tumors decreased significantly before and after NAC, as shown in Figure 3 and Table 5. ∆APTw values were significantly lower in non-MHR tumors compared with MHR tumors. (0.58 ± 0.98% and 1.44 ± 1.11%, respectively, *p* = 0.015) (Figure 4).

**Table 5:** Quantitative MRI data (mean ± standard deviation).

Variable	MHR (*n* = 14)	n-MHR (*n* = 36)	*P*
APT0 (%)	2.66 ± 0.50	3.13 ± 0.77	0.034*
APT1 (%)	1.23 ± 0.86	2.51 ± 0.98	<0.001*
ΔAPT (%)	1.44 ± 1.11	0.58 ± 1.17	0.015*
Dmax0 (mm)	53.02 ± 22.61	40.72 ± 22.90	0.093
Dmax1 (mm)	30.41 ± 16.91	31.95 ± 16.81	0.773
ΔDmax (mm)	22.61 ± 22.44	8.77 ± 10.97	0.005*
V0 (mm^3^)	24.26 ± 30.18	19.47 ± 21.74	0.535
V1 (mm^3^)	3.44 ± 3.17	7.04 ± 3.44	0.085
ΔV (mm^3^)	20.81 ± 29.58	12.39 ± 16.25	0.203
ADC0	1.03 ± 0.12	0.97 ± 0.25	0.038*
ADC1	1.15 ± 0.14	1.04 ± 0.20	0.035*
ΔADC	−0.18 ± 0.22	−0.17 ± 0.27	0.324

**Figure 5:**
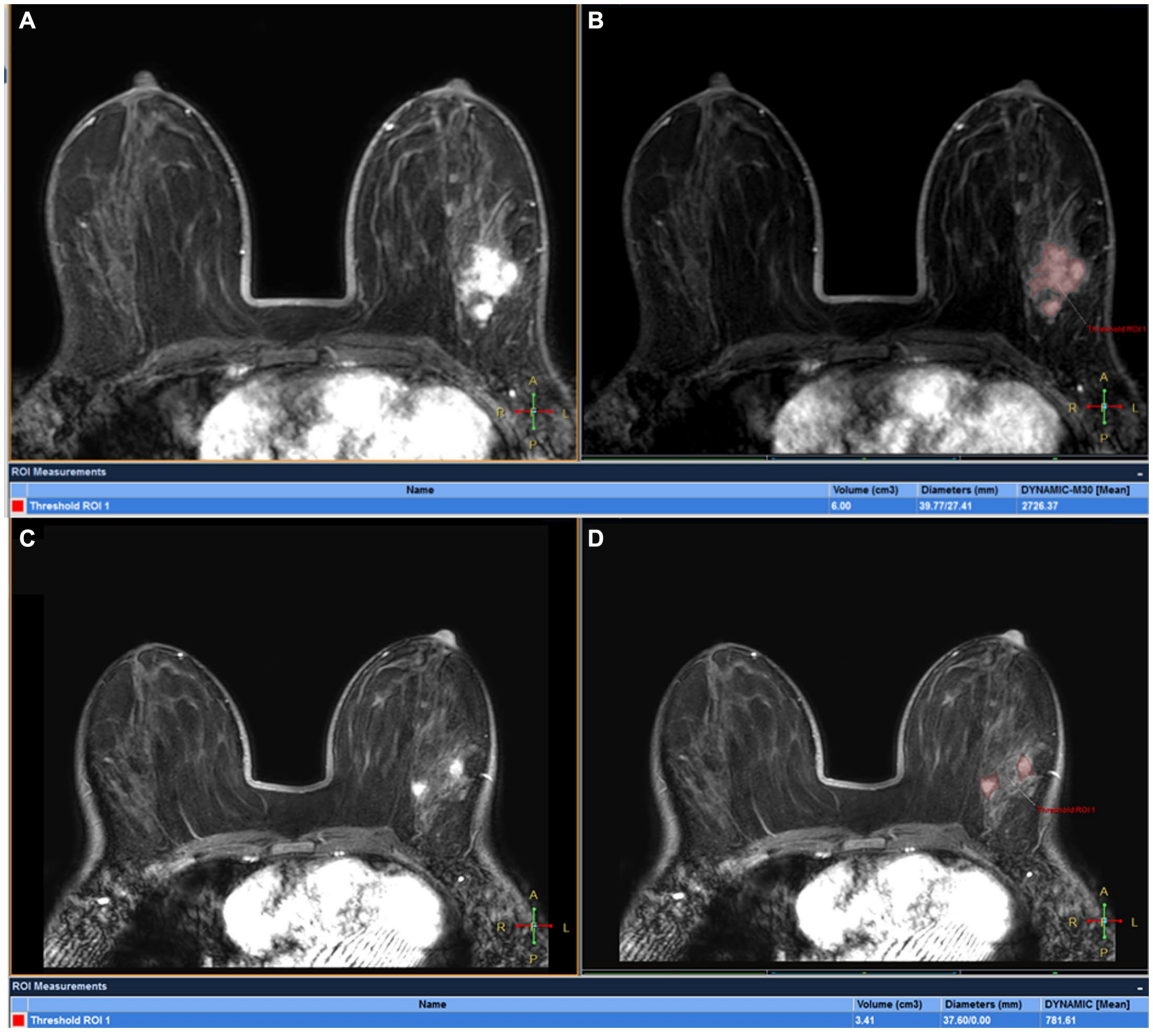
DCE-MRI of a 32-year-old woman with left ER+ invasive breast cancer at T0 **(A,B)** and T1 **(C,D)**: tumor maxmium diameter meausred at T0 (Dmax0) and T1 (Dmax1) were 39.77 and 37.60 mm, respectively and tumor volume meausred at T0(V0) and T1(V1) were 6.00 cm3 and 3.41 cm3, respectively. The patient was confirmed by pathological analysis of the surgical specimen collected after first 2 cycles of NAC as having pCR.

Comparison of Dmax V, and ADC values between the two groups is also shown in Figure 5; Table 5. ∆Dmax values were significantly higher in MHR tumors compared with non-MHR tumors (*p* = 0.005). ADC0 and ADC1 values were significantly higher in MHR tumors than in non-MHR tumors (*p* = 0.038 and 0.035). There was no significant difference in Dmax0, Dmax1, V0, V1, ∆V, and ∆ADC between the two groups (*p* = 0.324).

### Diagnostic efficacy

**Figure 6:**
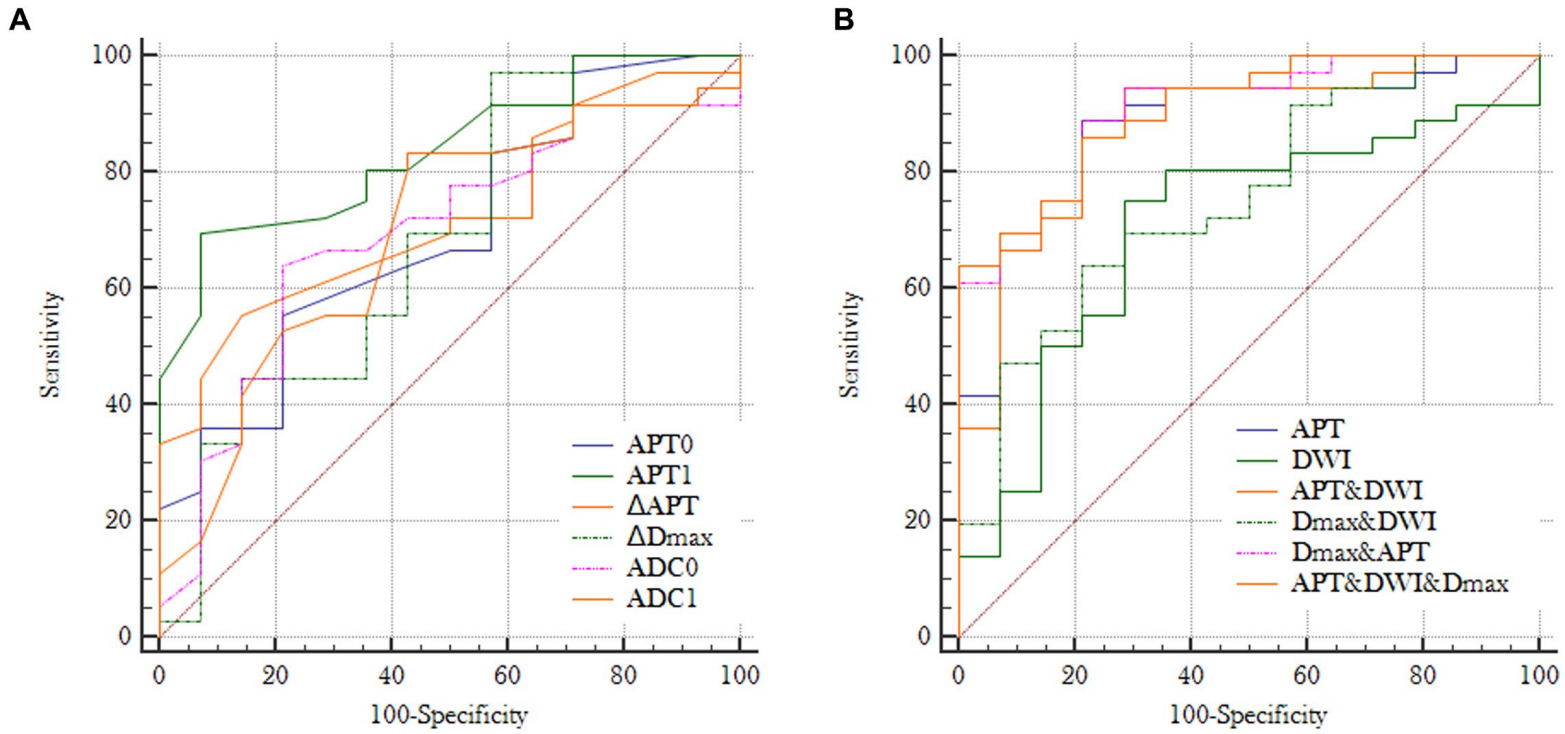
**(A)** Receiver operating characteristic curves for individual imaging parameters obtained at T0 or T1: AUC (APT0) = 0.690, AUC (ADC0) = 0.694, AUC (APT1) = 0.837, AUC (ΔAPTw) = 0.723, AUC (ΔDmax) = 0.685 and AUC (ADC1) = 0.692; **(B)** Receiver operating characteristic curves for different combinations of imaging paramenters: AUC (APT) = 0.879, AUC (DWI) = 0.704, AUC (Dmax+DWI) = 0.752, AUC (Dmax+APTw) = 0.903, AUC (APTw+DWI) = AUC (Dmax+DWI + APTw) = 0.904 (APT = APT0 + APT1 + ΔAPT; DWI = ADC0 + ADC1).

Before NAC (T0), areas under the curves (AUCs) for MHR prediction (Figure 6A) were acquired with optimal thresholds for APT0 and ADC0 of 0.690 and 0.694, respectively, (sensitivities of 55.6 and 63.9%, and specificities of 78.6 and 78.6%, respectively). At the end of the second cycle of NAC (T1), AUCs for MHR prediction (Figure 6A) were acquired with optimal thresholds for APT1, ∆APTw, ∆Dmax, and ADC1 of 0.837, 0.723, 0.685, and 0.692; sensitivities of 69.4, 55.6, 97.2, and 83.3% were obtained, respectively; and specificities of 92.9, 85.7, 42.9, and 57.1%, respectively. Predicting the efficacy of NAC in breast cancer, AUCs for MHR prediction (Figure 6B) were acquired using APT (APT0 + APT1 + ∆APT), DWI (ADC0&ADC1), ∆Dmax, ∆Dmax&DWI, ∆Dmax&APTw, and ∆Dmax&DWI&APTw with optimal thresholds of 0.879, 0.704, 0.685, 0.752, 0.903, and 0.903, respectively; sensitivities of 88.9, 75, 97.2%, 63.9, 88.9, and 86.1% were obtained, respectively, with specificities of 78.6, 71.4, 42.9%, 78.6, 78.6, and 78.6%, respectively. The Delong test showed AUC (Dmax+DWI + APTw) = AUC (Dmax+APTw) > AUC (APTw) > AUC (Dmax+DWI) > AUC (Dmax) (all *p* < 0.05).

## Discussion

This study demonstrated that high sensitivity performance can be achieved by APTw for early prediction of MHR status at the end of the first two NAC cycles, which might allow timely regimen refinement before definitive surgical treatment. APTw in combination with tumor diameter and DWI can further improve diagnostic accuracy.

Our previous studies confirmed that APTw values significantly differed between fibroadenomas and malignant breast tumors (18), and the repeatability and stability of 3D APTw were tested with good results. Higher field strength (7 T) may help increase the signal-to-noise and contrast-to-noise ratios in APTw imaging, and prolonged endogenous T1 relaxation under high field strength may also increase CEST signals (20). However, the high field intensity can increase the magnetic susceptibility and local field inhomogeneity of tissues (27), which may downgrade the performance of APTw imaging. Contrasting a previous study (19, 28) (using Dixon acquisition for water-fat separation), multi-echo Dixon acquisition was introduced for B0 map generation and thus B0 correction in the present study. Besides, SPIR (spectral pre-saturation with inversion recovery) but not SPAIR (spectral attenuated inversion recovery) was applied for fat suppression in APTw imaging in the present study for its better compatibility with the saturation pulses of APTw and shorter scan time. A previous study showed that due to high blood hemoglobin and albumin, angiogenesis increases protein contents (29) and thus elevates APTw values in malignant tumors. Additionally, in a study (17) assessing the effect of menstrual cycle on APT, menstrual cycle-related APT signal fluctuations seemed to be negligible compared to APT signal increase in breast cancer tissue. To avoid such effects, this study performed MR examination during 7–14 days of the menstrual cycle. Unilateral APTw imaging of only one breast instead of both breasts was performed to reduce the effects of B1 field inhomogeneity, with motion artifacts of the breast and scan time both considered in clinical application.

About 37% of patients exhibit no benefits from NAC (3). Early and accurate prediction of tumor response to NAC is critical for treatment management. Studies have revealed the MHR status or minimal residual disease as the best predictor of good long-term prognosis (30, 31). As the gold standard for evaluating tumor response, pathological examination has high diagnostic accuracy, but must be performed after surgery, making it easy to miss the best opportunity for treatment adjustment. Therefore, it is necessary to develop tools that could dynamically assess tumor response to NAC *in vivo*. Due to elevated hemoglobin and albumin concentrations, angiogenesis may increase protein signaling in malignant tumors (16, 29), indicating NAC-induced angionecrosis may potentially be examined by APTw. Dula et al. (16) evaluated only one patient by pCR status, whose APTw value decreased from 4.86 ± 0.15% to 3.5 ± 1.59% after 1 cycle of NAC. In this study, APTw values of MHR in 50 breast cancer patients significantly lower than non-MHR before NAC, and early after chemotherapy (2 cycles) were compared and it was found that APTw values in the MHR group decreased significantly after NAC, with a more pronounced reduction than in the non-MHR group. This observation corroborated a study by Zhang et al. (32) in which APTw values after NAC were significantly different from baseline in the MHR group (3.19% vs. 2.43%; *p* = 0.03), while there was no difference in the non-MHR group (2.76% vs. 2.50%, *p* > 0.05). These results indicate that NAC reduces mobile protein concentrations in MHR and non-MHR patients in early treatment, reflecting reduced protein expression in breast cancer cells stressed by NAC. Importantly, NAC-induced decrease in APTw value was not due to treatment-induced changes in tumor acidosis. In aggressive tumors, glycolysis upregulation is common, resulting in acidic tumors (33). The chemical exchange of amide protons with water is base-catalyzed; therefore, acidic tumors have low APTw values, which may be increased by therapies reducing glycolytic metabolism. We observed no increase in APTw in response to NAC, suggesting NAC-induced decrease in APTw is due to decreased mobile protein content rather than reduced glycolytic metabolism and tumor acidity. This finding corroborated a previous study (34).

The combination of DWI and DCE-MRI may improve the accuracy of differential diagnosis between benign and malignant tumors. DWI reflects the change of tumor cell density, which is more accurate than the change of tumor size in assessing NAC efficacy (35). ADC values derived from DWI may be a sensitive measure of the response of the cellular microenvironment to cytotoxic drugs. Park et al. (36) reported that cancers with low-ADC values on pretreatment images have good response to NAC. This study also demonstrated that ADC value can be used to predict NAC efficacy, consistent with two studies that found significantly higher pretreatment ADC values in responders compared with the non-MHR group (36, 37). In addition, lower pre-treatment ADC metrics were generally detected in responders to therapy in this study, in agreement with Wilmes et al. (38). However, ΔADC values did not differ between the two groups. Better results might be obtained with ADC values evaluated at an earlier time point. ADC evaluation is not stable in sensitivity to reflect NAC efficacy in the 2nd cycle may be too late to detect necrotic changes induced by chemotherapy, when lesions may have started to be transformed into fibrous tissue (39). Besides, because of no standardization of DWI acquisition, data post-processing and b value selection (0–800 s/mm^2^), divergent ΔADC data for evaluating the response to NAC have been reported (40–42). The scanning scheme of APTw has been proved to be relatively stable in the earlier researchs (16), and the measurement method is relatively uniform (14).

Tumor response to treatment was assessed with RECIST criteria based on the longest diameter of the target lesion, which has limitations, especially in tumor evaluation in the non-concentric shrinkage mode (NCS) (10, 43). In the present study, the threshold method was utilized to extract the volume of the whole breast tumor, and the maximum diameter of the tumor was assessed in three dimensions, which reduces errors caused by two-dimensional measurements and increases the accuracy of measurements. Lorenzon et al. (44) showed that volume measurement is very accurate for non-mass lesions. Rieber et al. (45) found that tumors may have no or weak enhancement after NAC, resulting in unreliable determination of residual tumor size in carcinomas with significant response to chemotherapy, which might lead to false-negative results. In the current study, the maximum diameters and volumes of tumors were measured by semi-automatic segmentation and fusion (22). As shown above, treatment evaluation using the maximum diameter of the tumor yielded similar efficiency to that of APTw. We found a significant difference in Dmax between the two groups, with a high diagnostic power for ∆Dmax. Hylton et al. (46) found the segmented volume performed better in MHR prediction after the first cycle than tumor diameter; however, in this study, diameter measures were more advantageous than or similar to tumor volume, corroborating a previous report by Minarikova et al. (39). Therefore, we speculated that APTw imaging can reflect the pathological changes of breast cancer earlier than morphological findings and help predict NAC efficacy earlier. The results showed that APTw change was more sensitive and appeared earlier than volume change. Additionally, APTw combined with Dmax without DWI improved the predictive efficacy of NAC to 90.3%. Although the combination of APTw and DWI as well as the maximum tumor diameter may provide a relatively high diagnostic efficiency (AUC = 0.903), there were still two mis-classified cases in this study (MHR mis-classified as non-MHR, including one triple-negative and one HER2-negative cases). We considered that differences in receptor status and Dmax (20–25 mm) led to prediction failure. APTw imaging for different molecular types of breast cancer needs further investigation.

## Limitations

There were limitations in this study. First, this study was a single-center investigation with a limited number of patients, especially MHR cases. Secondly, the effects of patient age, tumor size and extended molecular typing were not analyzed, and different NAC regimens were used, although all NAC regimens were confirmed to be standard. Finally, all patients in the prospective study had mass enhancement lesions, and non-mass enhancement lesions should be included in future studies.

## Conclusion

In summary, APTw value has potential diagnostic value in distinguishing between MHRs and non-MHRs. Specifically, APTw may be an early indicator of inferior response to NAC, enabling the discontinuation of ineffective treatment and the initiation of a more promising alternative. These findings suggest APTw MRI has good potential to evaluate and predict NAC efficacy.

## Data availability statement

The datasets presented in this study can be found in online repositories. The names of the repository/repositories and accession number(s) can be found in the article/Supplementary material.

## Ethics statement

The studies involving humans were approved by the Ethics Committee of First Affiliated Hospital of Dalian Medical University (PJ-KS-XJS-2020-19). The studies were conducted in accordance with the local legislation and institutional requirements. Written informed consent for participation in this study was provided by the participants’ legal guardians/next of kin. Written informed consent was obtained from the individual(s), and minor(s)’ legal guardian/next of kin, for the publication of any potentially identifiable images or data included in this article.

## Author contributions

NZ: Writing – original draft. QS: Writing – review & editing. HL: Data curation, Writing – review & editing. ZW: Formal analysis, Writing – review & editing. QW: Data curation, Writing – review & editing. HZ: Methodology, Writing – review & editing. LZ: Funding acquisition, Supervision, Visualization, Writing – review & editing. AL: Visualization, Writing – review & editing. HW: Resources, Writing – review & editing. JW: Supervision, Visualization, Writing – review & editing. LL: Supervision, Visualization, Writing – review & editing.

## References

[1] Hayes DF Schott AF. Neoadjuvant chemotherapy: what are the benefits for the patient and for the investigator? J Natl Cancer Inst Monogr. (2015) 2015:36–9. doi: 10.1093/jncimonographs/lgv004 26063884

[2] Tudorica A Oh KY Chui SY Roy N Troxell ML Naik A. Early prediction and evaluation of breast Cancer response to neoadjuvant chemotherapy using quantitative DCE-MRI. Transl Oncol. (2016) 9:8–17. doi: 10.1016/j.tranon.2015.11.016, PMID: 26947876 PMC4800060

[3] Haque W Verma V Hatch S Suzanne Klimberg V Brian Butler E Teh BS. Response rates and pathologic complete response by breast cancer molecular subtype following neoadjuvant chemotherapy. Breast Cancer Res Treat. (2018) 170:559–67. doi: 10.1007/s10549-018-4801-3 29693228

[4] Romeo V Accardo G Perillo T Basso L Garbino N Nicolai E. Assessment and prediction of response to neoadjuvant chemotherapy in breast Cancer: a comparison of imaging modalities and future perspectives. Cancers. (2021) 13:13. doi: 10.3390/cancers13143521 PMC8303777 34298733

[5] Telli ML Gradishar WJ Ward JH. NCCN guidelines updates: breast Cancer. J Natl Compr Cancer Netw. (2019) 17:552–5. doi: 10.6004/jnccn.2019.5006, PMID: 31117035

[6] Sun R Meng Z Hou X Chen Y Yang Y Huang G. Prediction of breast cancer molecular subtypes using DCE-MRI based on CNNs combined with ensemble learning. Phys Med Biol. (2021) 66:175009. doi: 10.1088/1361-6560/ac195a, PMID: 34330117

[7] Kim Y Kim SH Lee HW Song BJ Kang BJ Lee A. Intravoxel incoherent motion diffusion-weighted MRI for predicting response to neoadjuvant chemotherapy in breast cancer. Magn Reson Imaging. (2018) 48:27–33. doi: 10.1016/j.mri.2017.12.018 29278762

[8] Zhang D Geng X Suo S Zhuang Z Gu Y Hua J. The predictive value of DKI in breast cancer: does tumour subtype affect pathological response evaluations? Magn Reson Imaging. (2022) 85:28–34. doi: 10.1016/j.mri.2021.10.013 34662700

[9] Bayoumi D Zaky M Ibrahim DA Abdallah A Abouelkhair KM. The additive role of (1)H-magnetic resonance spectroscopic imaging to ensure pathological complete response after neoadjuvant chemotherapy in breast cancer patients. Pol J Radiol. (2019) 84:570–80. doi: 10.5114/pjr.2019.92282 PMC7016493 32082456

[10] Zhuang X Chen C Liu Z Zhang L Zhou X Cheng M. Multiparametric MRI-based radiomics analysis for the prediction of breast tumor regression patterns after neoadjuvant chemotherapy. Transl Oncol. (2020) 13:100831. doi: 10.1016/j.tranon.2020.100831, PMID: 32759037 PMC7399245

[11] Cho GY Gennaro L Sutton EJ Zabor EC Zhang Z Giri D. Intravoxel incoherent motion (IVIM) histogram biomarkers for prediction of neoadjuvant treatment response in breast cancer patients. Eur J Radiol Open. (2017) 4:101–7. doi: 10.1016/j.ejro.2017.07.002, PMID: 28856177 PMC5565789

[12] Fardanesh R Marino MA Avendano D Leithner D Pinker K Thakur SB. Proton MR spectroscopy in the breast: technical innovations and clinical applications. J Magn Reson Imaging. (2019) 50:1033–46. doi: 10.1002/jmri.26700, PMID: 30848037 PMC6732054

[13] van Zijl PCM Lam WW Xu J Knutsson L Stanisz GJ. Magnetization transfer contrast and chemical exchange saturation transfer MRI. Features and analysis of the field-dependent saturation spectrum. NeuroImage. (2018) 168:222–41. doi: 10.1016/j.neuroimage.2017.04.045, PMID: 28435103 PMC5650949

[14] Togao O Keupp J Hiwatashi A Yamashita K Kikuchi K Yoneyama M. Amide proton transfer imaging of brain tumors using a self-corrected 3D fast spin-echo dixon method: comparison with separate B(0) correction. Magn Reson Med. (2017) 77:2272–9. doi: 10.1002/mrm.26322, PMID: 27385636

[15] Zhou J Hong X Zhao X Gao JH Yuan J. APT-weighted and NOE-weighted image contrasts in glioma with different RF saturation powers based on magnetization transfer ratio asymmetry analyses. Magn Reson Med. (2013) 70:320–7. doi: 10.1002/mrm.24784, PMID: 23661598 PMC3723702

[16] Dula AN Arlinghaus LR Dortch RD Dewey BE Whisenant JG Ayers GD. Amide proton transfer imaging of the breast at 3 T: establishing reproducibility and possible feasibility assessing chemotherapy response. Magn Reson Med. (2013) 70:216–24. doi: 10.1002/mrm.24450, PMID: 22907893 PMC3505231

[17] Loi L Goerke S Zimmermann F Korzowski A Meissner JE Breitling J. Assessing the influence of the menstrual cycle on APT CEST-MRI in the human breast. Magn Reson Imaging. (2022) 91:24–31. doi: 10.1016/j.mri.2022.05.006, PMID: 35550841

[18] Zhang N Kang J Wang H Liu A Miao Y Ma X. Differentiation of fibroadenomas versus malignant breast tumors utilizing three-dimensional amide proton transfer weighted magnetic resonance imaging. Clin Imaging. (2022) 81:15–23. doi: 10.1016/j.clinimag.2021.09.002, PMID: 34597999

[19] Crescenzi R Donahue PMC Mahany H Lants SK Donahue MJ. CEST MRI quantification procedures for breast cancer treatment-related lymphedema therapy evaluation. Magn Reson Med. (2020) 83:1760–73. doi: 10.1002/mrm.28031, PMID: 31631410 PMC6982565

[20] Krikken E Khlebnikov V Zaiss M Jibodh RA van Diest PJ Luijten PR. Amide chemical exchange saturation transfer at 7 T: a possible biomarker for detecting early response to neoadjuvant chemotherapy in breast cancer patients. Breast Cancer Res. (2018) 20:51. doi: 10.1186/s13058-018-0982-2, PMID: 29898745 PMC6001024

[21] Fagan AJ Bitz AK Bjorkman-Burtscher IM Collins CM Kimbrell V Raaijmakers AJE. 7T MR safety. J Magn Reson Imaging. (2021) 53:333–46. doi: 10.1002/jmri.27319, PMID: 32830900 PMC8170917

[22] Sartoretti E Sartoretti T Wyss M Becker AS Schwenk A van Smoorenburg L. Amide proton transfer weighted imaging shows differences in multiple sclerosis lesions and White matter Hyperintensities of presumed vascular origin. Front Neurol. (2019) 10:1307. doi: 10.3389/fneur.2019.01307 31920930 PMC6914856

[23] Onishi N Sadinski M Hughes MC Ko ES Gibbs P Gallagher KM. Ultrafast dynamic contrast-enhanced breast MRI may generate prognostic imaging markers of breast cancer. Breast Cancer Res. (2020) 22:58. doi: 10.1186/s13058-020-01292-9, PMID: 32466799 PMC7254650

[24] Fusco R Di Marzo M Sansone C Sansone M Petrillo A. Breast DCE-MRI: lesion classification using dynamic and morphological features by means of a multiple classifier system. Eur Radiol Exp. (2017) 1:10. doi: 10.1186/s41747-017-0007-4, PMID: 29708202 PMC5909352

[25] Ogston KN Miller ID Payne S Hutcheon AW Sarkar TK Smith I. A new histological grading system to assess response of breast cancers to primary chemotherapy: prognostic significance and survival. Breast. (2003) 12:320–7. doi: 10.1016/S0960-9776(03)00106-1, PMID: 14659147

[26] Yu Y Jiang Q Miao Y Li J Bao S Wang H. Quantitative analysis of clinical dynamic contrast-enhanced MR imaging for evaluating treatment response in human breast cancer. Radiology. (2010) 257:47–55. doi: 10.1148/radiol.10092169, PMID: 20713609 PMC2941722

[27] Venkatesh V Sharma N Singh M. Intensity inhomogeneity correction of MRI images using InhomoNet. Comput Med Imaging Graph. (2020) 84:101748. doi: 10.1016/j.compmedimag.2020.101748, PMID: 32679471

[28] Zimmermann F Korzowski A Breitling J Meissner JE Schuenke P Loi L. A novel normalization for amide proton transfer CEST MRI to correct for fat signal-induced artifacts: application to human breast cancer imaging. Magn Reson Med. (2020) 83:920–34. doi: 10.1002/mrm.27983, PMID: 31532006

[29] Jiang S Yu H Wang X Lu S Li Y Feng L. Molecular MRI differentiation between primary central nervous system lymphomas and high-grade gliomas using endogenous protein-based amide proton transfer MR imaging at 3 tesla. Eur Radiol. (2016) 26:64–71. doi: 10.1007/s00330-015-3805-1 25925361 PMC4627862

[30] Kaufmann M von Minckwitz G Mamounas EP Cameron D Carey LA Cristofanilli M. Recommendations from an international consensus conference on the current status and future of neoadjuvant systemic therapy in primary breast cancer. Ann Surg Oncol. (2012) 19:1508–16. doi: 10.1245/s10434-011-2108-2, PMID: 22193884

[31] Bear HD Anderson S Smith RE Geyer CE Jr Mamounas EP Fisher B. Sequential preoperative or postoperative docetaxel added to preoperative doxorubicin plus cyclophosphamide for operable breast Cancer: National Surgical Adjuvant Breast and bowel project protocol B-27. J Clin Oncol. (2006) 24:2019–27. doi: 10.1200/JCO.2005.04.1665 16606972

[32] Zhang S Rauch GM Adrada BE Boge M Mohamed RMM Abdelhafez AH. Assessment of early response to neoadjuvant systemic therapy in triple-negative breast Cancer using amide proton transfer-weighted chemical exchange saturation transfer MRI: a pilot study. Radiol Imag Cancer. (2021) 3:e200155. doi: 10.1148/rycan.2021200155 PMC8489465 34477453

[33] Chen LQ Pagel MD. Evaluating pH in the extracellular tumor microenvironment using CEST MRI and other imaging methods. Adv Radiol. (2015) 2015:1–25. doi: 10.1155/2015/206405, PMID: 27761517 PMC5066878

[34] Krikken E van der Kemp WJM Khlebnikov V van Dalen T Los M van Laarhoven HWM. Contradiction between amide-CEST signal and pH in breast cancer explained with metabolic MRI. NMR Biomed. (2019) 32:e4110. doi: 10.1002/nbm.4110, PMID: 31136039 PMC6772111

[35] Le Bihan D Breton E Lallemand D Grenier P Cabanis E Laval-Jeantet M. MR imaging of intravoxel incoherent motions: application to diffusion and perfusion in neurologic disorders. Radiology. (1986) 161:401–7. doi: 10.1148/radiology.161.2.3763909, PMID: 3763909

[36] Park SH Moon WK Cho N Song IC Chang JM Park IA. Diffusion-weighted MR imaging: pretreatment prediction of response to neoadjuvant chemotherapy in patients with breast cancer. Radiology. (2010) 257:56–63. doi: 10.1148/radiol.10092021 20851939

[37] Iacconi C Giannelli M Marini C Cilotti A Moretti M Viacava P. The role of mean diffusivity (MD) as a predictive index of the response to chemotherapy in locally advanced breast cancer: a preliminary study. Eur Radiol. (2010) 20:303–8. doi: 10.1007/s00330-009-1550-z, PMID: 19760422

[38] Onishi N Li W Newitt DC Harnish RJ Strand F Nguyen AA. Breast MRI during neoadjuvant chemotherapy: lack of background parenchymal enhancement suppression and inferior treatment response. Radiology. (2021) 301:295–308. doi: 10.1148/radiol.2021203645, PMID: 34427465 PMC8574064

[39] Minarikova L Bogner W Pinker K Valkovic L Zaric O Bago-Horvath Z. Investigating the prediction value of multiparametric magnetic resonance imaging at 3 T in response to neoadjuvant chemotherapy in breast cancer. Eur Radiol. (2017) 27:1901–11. doi: 10.1007/s00330-016-4565-2, PMID: 27651141 PMC5374186

[40] Eun NL Kang D Son EJ Park JS Youk JH Kim JA. Texture analysis with 3.0-T MRI for Association of Response to neoadjuvant chemotherapy in breast Cancer. Radiology. (2020) 294:31–41. doi: 10.1148/radiol.2019182718, PMID: 31769740

[41] Ramirez-Galvan YA Cardona-Huerta S Elizondo-Riojas G Alvarez-Villalobos NA. Apparent diffusion coefficient value to evaluate tumor response after neoadjuvant chemotherapy in patients with breast Cancer. Acad Radiol. (2018) 25:179–87. doi: 10.1016/j.acra.2017.08.009 29033147

[42] Du S Gao S Zhao R Liu H Wang Y Qi X. Contrast-free MRI quantitative parameters for early prediction of pathological response to neoadjuvant chemotherapy in breast cancer. Eur Radiol. (2022) 32:5759–72. doi: 10.1007/s00330-022-08667-w, PMID: 35267091

[43] Van Persijn van Meerten EL Gelderblom H Bloem JL. RECIST revised: implications for the radiologist. A review article on the modified RECIST guideline. Eur Radiol. (2010) 20:1456–67. doi: 10.1007/s00330-009-1685-y, PMID: 20033179 PMC2872013

[44] Lorenzon M Zuiani C Londero V Linda A Furlan A Bazzocchi M. Assessment of breast cancer response to neoadjuvant chemotherapy: is volumetric MRI a reliable tool? Eur J Radiol. (2009) 71:82–8. doi: 10.1016/j.ejrad.2008.03.021 18472240

[45] Rieber A Brambs HJ Gabelmann A Heilmann V Kreienberg R Kuhn T. Breast MRI for monitoring response of primary breast cancer to neo-adjuvant chemotherapy. Eur Radiol. (2002) 12:1711–9. doi: 10.1007/s00330-001-1233-x, PMID: 12111062

[46] Hylton NM Blume JD Bernreuter WK Pisano ED Rosen MA Morris EA. Locally advanced breast Cancer: MR imaging for prediction of response to neoadjuvant chemotherapy—Results from ACRIN 6657/I-SPY TRIAL. Radiology. (2012) 263:663–72. doi: 10.1148/radiol.12110748, PMID: 22623692 PMC3359517

