# Supplementary material for: Early prediction of pathological response to neoadjuvant chemotherapy of breast tumors: a comparative study using amide proton transfer-weighted, diffusion weighted and dynamic contrast enhanced MRI
Source: Front Med (Lausanne). 2024 Jan 17;11:1295478. doi: 10.3389/fmed.2024.1295478 (PMC10827983; doi:10.3389/fmed.2024.1295478)
Supplement: Supplementary file 1 [file Data_Sheet_1.doc]

**Supplementary material**

Supplementary Table 1

| Grade 1 | No change or some alteration to individual malignant cells but no reduction in overall cellularity |
| --- | --- |
| Grade 2 | A minor loss of tumour cells but overall cellularity still high; up to 30% loss |
| Grade 3 | Between an estimated 30% and 90% reduction in tumour cells |
| Grade 4 | A marked disappearance of tumour cells such that only small clusters or widely dispersed individual cells remain; more than 90% loss of tumour cells |
| Grade 5 | No malignant cells identifiable in sections from the site of the tumour; only vascular fibroelastotic stroma remains often containing macrophages. However, ductal carcinoma in situ (DCIS) may be present |

Supplementary Table 2（measurement data by reader 1 and reader2）

| **APT0** | **APT1** | **△APT** | **D0** | **D1** | **ΔD** | **V0** | **V1** | **ΔV** |
| --- | --- | --- | --- | --- | --- | --- | --- | --- |
|  |  |  |  |  |  |  |  |  |
| 2.4 | 3.9 | -1.5 | 68.92 | 74.85 | -5.93 | 33.18 | 11.08 | 22.1 |
| 3.7 | 3.1 | 0.6 | 82.32 | 52.99 | 29.33 | 89.32 | 23.31 | 66.01 |
| -1.6 | -7.1 | 5.5 | 43.03 | 29.22 | 13.81 | 5.5 | 2.73 | 2.77 |
| 2.5 | 2.4 | 0.1 | 39.81 | 18.39 | 21.42 | 12.55 | 2.07 | 10.48 |
| 3.7 | 0.9 | 2.8 | 35.5 | 16.4 | 19.1 | 12.92 | 1.337 | 11.583 |
| 3.4 | 3 | 0.4 | 23.34 | 20.2 | 3.14 | 9.76 | 8.23 | 1.53 |
| 3 | 4.8 | -1.8 | 15.7 | 12.41 | 3.29 | 0.72 | 0.62 | 0.1 |
| 4.9 | 3.6 | 1.3 | 39.77 | 37.6 | 2.17 | 6 | 3.41 | 2.59 |
| 2.8 | 1.9 | 0.9 | 72.88 | 51.8 | 21.08 | 30.74 | 3.59 | 27.15 |
| 4.8 | 1.2 | 3.6 | 83.52 | 68.66 | 14.86 | 51.88 | 8.45 | 43.43 |
| 3.4 | 3.2 | 0.2 | 85.04 | 58.47 | 26.57 | 60.62 | 29.96 | 30.66 |
| 3 | 2.6 | 0.4 | 26.87 | 28.1 | -1.23 | 3.84 | 3.53 | 0.31 |
| 3.2 | 2.4 | 0.8 | 78.58 | 68.1 | 10.48 | 35.65 | 15.22 | 20.43 |
| 2.5 | 3.1 | -0.6 | 25.2 | 22.5 | 2.7 | 2.614 | 1.369 | 1.245 |
| 2.4 | 3.7 | -1.3 | 54.4 | 35.6 | 18.8 | 55.808 | 12.826 | 42.982 |
| 2.1 | 3.2 | -1.1 | 26.9 | 19.3 | 7.6 | 3.447 | 2.562 | 0.885 |
| 5.3 | 2.1 | 3.2 | 24 | 16.9 | 7.1 | 4.52 | 3.43 | 1.09 |
| 3.1 | 1.2 | 1.9 | 3.4 | 1.9 | 1.5 | 0.54 | 0.21 | 0.33 |
| 5 | 3.4 | 1.6 | 27.85 | 20.63 | 7.22 | 4.6 | 0.73 | 3.87 |
| 2.2 | 1.8 | 0.4 | 35 | 33.8 | 1.2 | 6.273 | 6.042 | 0.231 |
| 1.7 | 2.5 | -0.8 | 40.3 | 34.1 | 6.2 | 16.281 | 12.699 | 3.582 |
| -0.7 | 0.1 | -0.8 | 39.4 | 33.5 | 5.9 | 4.454 | 4.357 | 0.097 |
| 2.1 | 2.6 | -0.5 | 16.2 | 41.23 | -25.03 | 45.34 | 10.79 | 34.55 |
| 1.6 | 1 | 0.6 | 60.02 | 28.3 | 31.72 | 33.3 | 3.39 | 29.91 |
| 0.9 | 0.2 | 0.7 | 23 | 11.1 | 11.9 | 4.489 | 0.817 | 3.672 |
| 2.1 | 0.9 | 1.2 | 41.4 | 39.9 | 1.5 | 18.309 | 13.342 | 4.967 |
| 1.5 | 1.3 | 0.2 | 12.6 | 12.2 | 0.4 | 0.74 | 0.336 | 0.404 |
| 2.1 | 2.1 | 0 | 28.58 | 24.27 | 4.31 | 5.83 | 1.58 | 4.25 |
| 2.5 | 2.6 | -0.1 | 35.33 | 27.08 | 8.25 | 5.2 | 1.23 | 3.97 |
| 2.5 | 2 | 0.5 | 57.88 | 46.6 | 11.28 | 19.88 | 9.68 | 10.2 |
| 2.8 | 3.3 | -0.5 | 28.2 | 23.8 | 4.4 | 2.439 | 1.335 | 1.104 |
| 2.9 | 1.5 | 1.4 | 63 | 41 | 22 | 37.808 | 11.356 | 26.452 |
| 2.3 | 2.9 | -0.6 | 77.07 | 62.43 | 14.64 | 51.33 | 26.65 | 24.68 |
| 2.2 | 0.9 | 1.3 | 28.1 | 27.2 | 0.9 | 9.272 | 6.816 | 2.456 |
| 2.5 | 2.7 | -0.2 | 2.9 | 3.4 | -0.5 | 1.21 | 1.45 | -0.24 |
| 7.6 | 8.1 | -0.5 | 28.1 | 28.2 | -0.1 | 5.241 | 4.554 | 0.687 |
| 1.8 | 1 | 0.8 | 33.1 | 27.3 | 5.8 | 6.461 | 2.874 | 3.587 |
| 4.6 | 1.8 | 2.8 | 25.5 | 23.2 | 2.3 | 4.485 | 2.662 | 1.823 |
| 0.6 | 0.3 | 0.3 | 63 | 41 | 22 | 37.808 | 11.356 | 26.452 |

measurement data by reader 2:

| **APT0** | **APT1** | **△APT** | **D0** | **D1** | **ΔD** | **V0** | **V1** | **ΔV** |
| --- | --- | --- | --- | --- | --- | --- | --- | --- |
|  |  |  |  |  |  |  |  |  |
| 2.4 | 3.9 | -1.5 | 68.92 | 74.85 | -5.93 | 33.18 | 11.08 | 22.1 |
| 3.7 | 3.1 | 0.6 | 82.32 | 52.99 | 29.33 | 89.32 | 23.31 | 66.01 |
| -1.6 | -7.1 | 5.5 | 43.03 | 29.22 | 13.81 | 5.5 | 2.73 | 2.77 |
| 2.5 | 2.4 | 0.1 | 39.81 | 18.39 | 21.42 | 12.55 | 2.07 | 10.48 |
| 3.7 | 0.9 | 2.8 | 35.5 | 16.4 | 19.1 | 12.92 | 1.337 | 11.583 |
| 3.4 | 3 | 0.4 | 23.34 | 20.2 | 3.14 | 9.76 | 8.23 | 1.53 |
| 3 | 4.8 | -1.8 | 15.7 | 12.41 | 3.29 | 0.72 | 0.62 | 0.1 |
| 4.9 | 3.6 | 1.3 | 39.77 | 37.6 | 2.17 | 6 | 3.41 | 2.59 |
| 2.8 | 1.9 | 0.9 | 72.88 | 51.8 | 21.08 | 30.74 | 3.59 | 27.15 |
| 4.8 | 1.2 | 3.6 | 83.52 | 68.66 | 14.86 | 51.88 | 8.45 | 43.43 |
| 3.4 | 3.2 | 0.2 | 85.04 | 58.47 | 26.57 | 60.62 | 29.96 | 30.66 |
| 3 | 2.6 | 0.4 | 26.87 | 28.1 | -1.23 | 3.84 | 3.53 | 0.31 |
| 3.2 | 2.4 | 0.8 | 78.58 | 68.1 | 10.48 | 35.65 | 15.22 | 20.43 |
| 2.5 | 3.1 | -0.6 | 25.2 | 22.5 | 2.7 | 2.614 | 1.369 | 1.245 |
| 2.4 | 3.7 | -1.3 | 54.4 | 35.6 | 18.8 | 55.808 | 12.826 | 42.982 |
| 2.1 | 3.2 | -1.1 | 26.9 | 19.3 | 7.6 | 3.447 | 2.562 | 0.885 |
| 5.3 | 2.1 | 3.2 | 24 | 16.9 | 7.1 | 4.52 | 3.43 | 1.09 |
| 3.1 | 1.2 | 1.9 | 3.4 | 1.9 | 1.5 | 0.54 | 0.21 | 0.33 |
| 5 | 3.4 | 1.6 | 27.85 | 20.63 | 7.22 | 4.6 | 0.73 | 3.87 |
| 2.2 | 1.8 | 0.4 | 35 | 33.8 | 1.2 | 6.273 | 6.042 | 0.231 |
| 1.7 | 2.5 | -0.8 | 40.3 | 34.1 | 6.2 | 16.281 | 12.699 | 3.582 |
| -0.7 | 0.1 | -0.8 | 39.4 | 33.5 | 5.9 | 4.454 | 4.357 | 0.097 |
| 2.1 | 2.6 | -0.5 | 16.2 | 41.23 | -25.03 | 45.34 | 10.79 | 34.55 |
| 1.6 | 1 | 0.6 | 60.02 | 28.3 | 31.72 | 33.3 | 3.39 | 29.91 |
| 0.9 | 0.2 | 0.7 | 23 | 11.1 | 11.9 | 4.489 | 0.817 | 3.672 |
| 2.1 | 0.9 | 1.2 | 41.4 | 39.9 | 1.5 | 18.309 | 13.342 | 4.967 |
| 1.5 | 1.3 | 0.2 | 12.6 | 12.2 | 0.4 | 0.74 | 0.336 | 0.404 |
| 2.1 | 2.1 | 0 | 28.58 | 24.27 | 4.31 | 5.83 | 1.58 | 4.25 |
| 2.5 | 2.6 | -0.1 | 35.33 | 27.08 | 8.25 | 5.2 | 1.23 | 3.97 |
| 2.5 | 2 | 0.5 | 57.88 | 46.6 | 11.28 | 19.88 | 9.68 | 10.2 |
| 2.8 | 3.3 | -0.5 | 28.2 | 23.8 | 4.4 | 2.439 | 1.335 | 1.104 |
| 2.9 | 1.5 | 1.4 | 63 | 41 | 22 | 37.808 | 11.356 | 26.452 |
| 2.3 | 2.9 | -0.6 | 77.07 | 62.43 | 14.64 | 51.33 | 26.65 | 24.68 |
| 2.2 | 0.9 | 1.3 | 28.1 | 27.2 | 0.9 | 9.272 | 6.816 | 2.456 |
| 2.5 | 2.7 | -0.2 | 2.9 | 3.4 | -0.5 | 1.21 | 1.45 | -0.24 |
| 7.6 | 8.1 | -0.5 | 28.1 | 28.2 | -0.1 | 5.241 | 4.554 | 0.687 |
| 1.8 | 1 | 0.8 | 33.1 | 27.3 | 5.8 | 6.461 | 2.874 | 3.587 |
| 4.6 | 1.8 | 2.8 | 25.5 | 23.2 | 2.3 | 4.485 | 2.662 | 1.823 |
| 0.6 | 0.3 | 0.3 | 63 | 41 | 22 | 37.808 | 11.356 | 26.452 |

**Supplementary Table 3 and Figures** (Results of Bland-Altman statistics)

| **Variable** | **Reader1** | **Reader2** | **ICC** |
| --- | --- | --- | --- |
| APT0(%) | 3.66±0.50 | 3.78±0.45 | 0.940 |
| APT1(%) | 2.23±0.86 | 2.56±0.77 | 0.921 |
| Dmax0(mm) | 43.02±20.61 | 43.66±20.99 | 0.997 |
| Dmax1(mm) | 30.41±16.91 | 30.56±16.45 | 0.940 |
| V0(mm^3^) | 28.26±30.90 | 28.89±30.59 | 0.992 |
| V1(mm^3^) | 5.44±3.17 | 5.45±3.78 | 0.996 |
| ADC0 | 1.12±0.17 | 1.55±0.17 | 0.940 |
| ADC1 | 1.05±0.11 | 1.05±0.77 | 0.921 |

Figure 1. APT0 of Bland-Altman statistics

Figure 2. APT1 of Bland-Altman statistics

Figure 3. D_max0_ of Bland-Altman statistics

Figure 4. D_max1_ of Bland-Altman statistics

Figure 5. V_0_ of Bland-Altman statistics

Figure 6. V1 of Bland-Altman statistics

Figure 7. ADC_0_ of Bland-Altman statistics

Figure 8. ADC1 of Bland-Altman statistics
